# P-176. Risk Factors for Non-Screening of Infectious Diseases Among Migrants in Nuevo Leon, Mexico

**DOI:** 10.1093/ofid/ofaf695.400

**Published:** 2026-01-11

**Authors:** Reynaldo Lara-Medrano, Victor Baylon-Valdez, Luis G Castillo-Reyna, Gloria Mayela Aguirre-García, María T Ramírez-Elizondo, Michel F Martínez-Reséndez, Francisco J Bosques-Padilla

**Affiliations:** School of Medicine and Health Sciences, Instituto Tecnológico y de Estudios Superiores de Monterrey, Monterrey, Nuevo Leon, Mexico; Instituto Tecnológico y de Estudios Superiores de Monterrey, School of Medicine and Health Sciences, Monterrey, Nuevo Leon, Mexico, Monterrey, Nuevo Leon, Mexico; School of Medicine and Health Sciences, Instituto Tecnológico y de Estudios Superiores de Monterrey, Monterrey, Nuevo Leon, Mexico; TecSalud, Monterrey, Nuevo Leon, Mexico; School of Medicine and Health Sciences, Instituto Tecnológico y de Estudios Superiores de Monterrey, Monterrey, Nuevo Leon, Mexico; Instituto Tecnológico y de Estudios Superiores de Monterrey, School of Medicine and Health Sciences, Monterrey, Nuevo Leon, Mexico, Monterrey, Nuevo Leon, Mexico; School of Medicine and Health Sciences, Instituto Tecnológico y de Estudios Superiores de Monterrey, Monterrey, Nuevo Leon, Mexico

## Abstract

**Background:**

Migrants represent a vulnerable population with unique barriers to healthcare access, including infectious disease screening. Despite recommendations for routine HIV, hepatitis B (HBV), and hepatitis C (HCV) screening in this population, testing rates remain suboptimal. Understanding the factors associated with non-screening is essential for developing targeted interventions.

Adjusted odds ratios for factors associated with non-screening for infectious diseases among migrants in transit.

**Figure:**
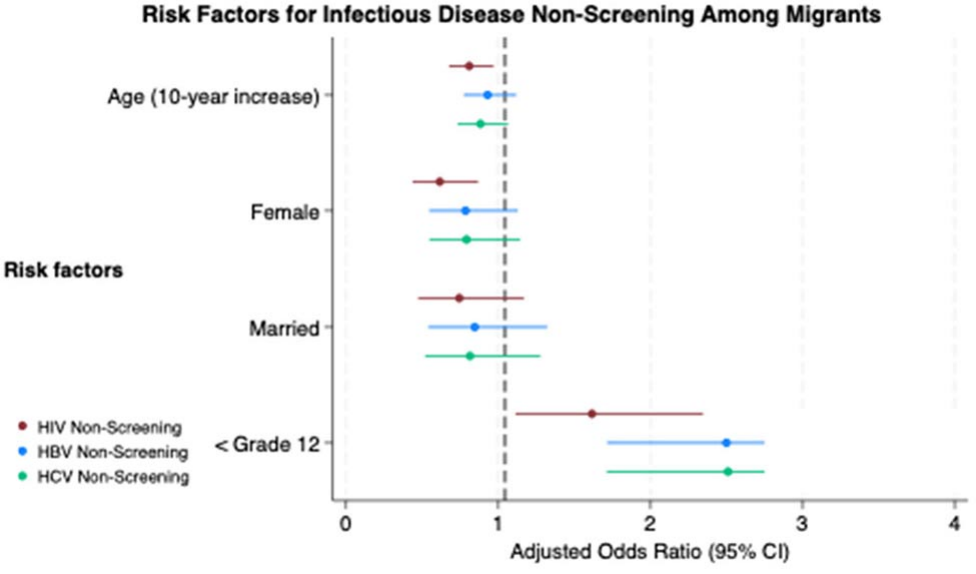

Lower educational attainment (< grade 12) was the strongest risk factor across all infections, with odds ratios ranging from 1.62 for HIV to 2.51 for HCV. Female gender and older age were protective against HIV non-screening only. Odds ratios adjusted for all variables shown.

**Methods:**

Community-based screening programs were conducted in shelters in the state of Nuevo Leon, Mexico. From December 2023 to December 2024, adults from Central and South America, transiting through Nuevo Leon to the United States, were invited to participate. A sociodemographic questionnaire was administered, and screening was performed using rapid tests for HIV, hepatitis B (HBV), and hepatitis C (HCV). Key variables analyzed included age, birth sex, marital status, and educational attainment. Separate multivariable logistic regression models were constructed for each infection to identify factors independently associated with lack of previous screening.

**Results:**

A total of 586 migrants participated, with 308 (53%) females and a mean age of 34 years (SD 10). Lower educational attainment was the most consistent risk factor for non-screening across all infections. Migrants with less than grade 12 education had significantly higher odds of never being screened for HIV (aOR 1.62, 95% CI: 1.11, 2.35), HBV (aOR 2.50, 95% CI: 1.71, 3.64), and HCV (aOR 2.51, 95% CI: 1.71, 3.68) compared to those with more than grade 12 education. Male gender was associated with higher odds of HIV non-screening (aOR 1.63, 95% CI: 1.15, 2.29). Older age was protective against HIV non-screening (aOR 0.81 per 10-year increase, 95% CI: 0.68, 0.97), while age did not significantly impact hepatitis non-screening. Marital status did not significantly affect non-screening rates for any infection.

**Conclusion:**

Lower educational attainment represents the most significant risk factor for infectious disease non-screening among migrants transiting through Mexico. Males and younger individuals are also at higher risk for HIV non-screening, specifically. Targeted interventions focused on migrants with lower educational levels, males, and younger individuals are needed to improve screening rates.

**Disclosures:**

All Authors: No reported disclosures

